# The Physiological Responses of *Escherichia coli* Triggered by Phosphoribulokinase (PrkA) and Ribulose-1,5-Bisphosphate Carboxylase/Oxygenase (Rubisco)

**DOI:** 10.3390/microorganisms8081187

**Published:** 2020-08-04

**Authors:** En-Jung Liu, I-Ting Tseng, Yi-Ling Chen, Ju-Jiun Pang, Zhi-Xuan Shen, Si-Yu Li

**Affiliations:** 1 Department of Chemical Engineering, National Chung Hsing University, Taichung City 40227, Taiwan.; kiwi_go@hotmail.com (E.-J.L.); haha541038@gmail.com (I.-T.T.); linda653199@gmail.com (Y.-L.C.); z4707090@gmail.com (J.-J.P.); s10121123@gmail.com (Z.-X.S.); 2 Innovation and Development Center of Sustainable Agriculture, National Chung Hsing University, Taichung City 40227, Taiwan

**Keywords:** glyoxylate shunt, methylglyoxal pathway, phosphoribulokinase (PrkA), redox balance, ribulose-1,5-bisphosphate carboxylase/oxygenase (Rubisco)

## Abstract

Phosphoribulokinase (PrkA) and ribulose-1,5-bisphosphate carboxylase/oxygenase (Rubisco) have been proposed to create a heterologous Rubisco-based engineered pathway in *Escherichia coli* for in situ CO_2_ recycling. While the feasibility of a Rubisco-based engineered pathway has been shown, heterologous expressions of PrkA and Rubisco also induced physiological responses in *E. coli* that may compete with CO_2_ recycling. In this study, the metabolic shifts caused by PrkA and Rubisco were investigated in recombinant strains where *ppc* and *pta* genes (encodes phosphoenolpyruvate carboxylase and phosphate acetyltransferase, respectively) were deleted from *E. coli* MZLF (*E. coli* BL21(DE3) Δ*zwf*, Δ*ldhA,* Δ*frd*). It has been shown that the demand for ATP created by the expression of PrkA significantly enhanced the glucose consumptions of *E. coli* CC (MZLF Δ*ppc*) and *E. coli* CA (MZLF Δ*ppc*, Δ*pta*). The accompanying metabolic shift is suggested to be the *mgsA* route (the methylglyoxal pathway) which results in the lactate production for reaching the redox balance. The overexpression of Rubisco not only enhanced glucose consumption but also bacterial growth. Instead of the *mgsA* route, the overproduction of the reducing power was balanced by the ethanol production. It is suggested that Rubisco induces a high demand for acetyl-CoA which is subsequently used by the glyoxylate shunt. Therefore, Rubisco can enhance bacterial growth. This study suggests that responses induced by the expression of PrkA and Rubisco will reach a new energy balance profile inside the cell. The new profile results in a new distribution of the carbon flow and thus carbons cannot be majorly directed to the Rubisco-based engineered pathway.

## 1. Introduction

The Calvin-Benson-Bassham (CBB) cycle widely exists in plants, microalgae, cyanobacteria, and some prokaryotes [1]. It is the primary pathway that is responsible for the carbon fixation in our ecosystem. The key enzyme of the CBB cycle is ribulose-1,5-bisphosphate carboxylase/oxygenase (Rubisco), which incorporates carbon dioxide into the second carbon of the phosphoryl-pentose backbone. Previous studies have shown that the eukaryotic form I Rubisco is difficult to be functionally expressed in *E. coli* [2,3], whereas the cyanobacterial forms I, II, and III Rubisco can be [4,5,6]. Phosphoribulokinase (PrkA) catalyzes phosphorylation of ribulose 5-phosphate (Ru5P) to form ribulose-1,5-bisphosphate (RuBP), which is the substrate of Rubisco. The phosphate donor is ATP.

In a previous study, the feasibility of Rubisco-based engineered *E. coli* for in situ CO_2_ recycling during the fermentation of pentoses was demonstrated [7]. By enhancing the function of native non-oxidative pentose phosphate pathway, Rubisco-based engineered *E. coli* can be further used to achieve a low CO_2_ emission during the fermentation of hexoses [8]. By constructing *E. coli* strain MZLF (*E. coli* BL21(DE3) Δ*zwf*, Δ*ldh*, Δ*frd*), in vivo activity of Rubisco-based engineered pathway can be estimated by analyzing the ratio of total C-2 (2-carbon fermentation products) to total C-1 (1-carbon fermentation product) [9]. Note that *zwf*, *ldh*, and *frd* genes encode glucose-6-phosphate 1-dehydrogenase, lactate dehydrogenase, and fumarate reductase, respectively. The other advantage of MZLF is that the carbon flow can be efficiently diverted to acetyl-CoA production, which is important to in situ CO_2_ recycling. By taking this advantage, we further achieved the performance of 2.17 mol/mol-glucose, which exceeded the theoretical yield of the conventional fermentation (2 mol/mol-glucose) [10].

On the other hand, the heterologous expressions of PrkA and Rubisco induced interesting physiological perturbations in *E. coli*. PrkA is usually considered as a toxic enzyme since the reaction product RuBP cannot be further metabolized in *E. coli* [5,6,7]. One important adverse effect is that a long lag phase (up to 10 h) is usually observed for the cultivation of *E. coli* that contains heterologous PrkA [7]. Nevertheless, *E. coli* can still find a way to detoxify the adverse effect caused by PrkA and have a normal growth when entering the log phase [7]. Furthermore, it has been shown that *E. coli* J1 [*E. coli* BL21(DE3) containing PrkA originated from *Synechococcus* PCC7942] had a better growth in LB medium when *L*-arabinose was used as the carbon source [7]. It can be summarized that the physiological responses induced by PrkA are allegedly associated with not only ATP imbalance but also cell growth.

The expression of Rubisco also exhibited profound physiological impacts. It is interesting to see that the overexpression of Rubisco in *E. coli* MZLF *(E. coli* BL21(DE3) Δ*zwf*, Δ*ldhA,* and Δ*frd*) greatly enhances the accumulation of pyruvate from 0.16 ± 0.12 to 0.46 ± 0.02 *mol/mol* [9]. This indicates that the chemical energy of glucose is not necessarily fully released. Furthermore, when pyruvate is converted to ethanol or acetate, the ethanol/acetate ratio is increased in the presence of Rubisco [9]. It is suggested that the overexpression of Rubisco perturbed the energy balance inside the cell [9]. When form I Rubisco was expressed in *E. coli*, an enhanced biomass accumulation was observed during conditions such as aerobic cultivation of *E. coli* BL21(DE3) using *D*-xylose [7], aerobic cultivation of *E. coli* BL21(DE3) using *L*-arabinose [7], anaerobic fermentation of *E. coli* BL21(DE3) using glucose (Li et al., 2015 and unpublished data), and anaerobic fermentation of *E. coli* MZLF using glucose (Yang et al., 2016 and unpublished data). This can be arguably attributed to the enhanced glyoxylate shunt based on transcriptome analysis [9]. While the glyoxylate shunt has shown to be enhanced in the *arcA* (encodes redox-dependent transcriptional activators ArcA) mutant [11], it has been previously shown that the transcriptional of *arcA* is down-regulated in *E. coli* in the presence of Rubisco [9]. It can be summarized that the metabolic perturbations caused by Rubisco are associated with bacterial growth and intracellular energy balance.

In order to precisely describe 1. the effect of ATP consumption catalyzed by PrkA on physiological perturbations and 2. enhanced bacterial growth and energy-rebalance caused by Rubisco, *E. coli* strains derived from MZLF were constructed where genes regarding bacterial growth and ATP formation were knocked out from MZLF strain. We herein proposed that Rubisco- or PrkA-induced physiological impacts may be fully revealed by creating a circumstance that is stressful for bacterial growth and energy production. In this manner, the enhanced cell growth (induced by Rubisco) and the energy rebalance (induced by PrkA and Rubisco) can be amplified. The use of MZLF as the parental strain in this study is for the consideration of the simplified metabolism. In this study, recombinant *E. coli* strains CC and CA with the deletion of *ppc* and with the double mutations of *ppc* and *pta* were respectively constructed from *E. coli* strain MZLF. The *ppc* gene encodes phosphoenolpyruvate carboxylase where phosphoenolpyruvate carboxylase converts phosphoenolpyruvate to oxaloacetate. It has been shown that the deletion of *ppc* is lethal for *E. coli* growth on glucose as the sole carbon source [12,13]. The acetate production through the *pta* (route (encoding phosphate acetyltransferase) is substrate-level phosphorylation to obtain ATP [14]. These two deletions will create significant stresses for *E. coli* so that the actual perturbations caused by PrkA and Rubisco can be significantly revealed in this harsh regime. The knowledge of the metabolic shifts caused by Rubisco and PrkA may further enhance the efficiency of the Rubisco-based engineered pathway for CO_2_ utilization.

## 2. Materials and Methods

### 2.1. Bacterial Strains and Plasmids

The strains and plasmids were listed in Table 1. To construct *E. coli* strain CC, the one-step inactivation procedure [15] was adopted to delete the *ppc* gene from the chromosome of *E. coli* MZLF [9]. Briefly speaking, the Q5 High-Fidelity DNA polymerase (New England Biolabs, Ipswich, MA, USA) and primers PPChf-HP1 and PPChr-HP2 (Table 2) were used to amplify FRT-*kan*^R^-FRT DNA fragment from pKD13 [15]. The linear DNA fragments were purified from the polymer chain reaction (PCR) mixture by subjecting them to the electrophoresis with the 0.8% agarose gel followed by the recovery of DNA using the Plus DNA Clean/Extraction Kit (GMbiolab Co, Ltd., Taichung, Taiwan). The purified DNA fragments were first treated with *Dpn*I followed by a two-step ethanol precipitation (70% ethanol). The precipitated DNA fragments were then transformed into *E. coli* MZLF/pKD46 through the electroporation (0.2-cm cuvette, 1.8 kV) (Bio-Rad Laboratories Inc, Philadelphia, PA, USA). Then, 15 g/L of L-arabinose was used to induce the expression of Gam, Bet, and Exo from pKD46 [15] for the homologous recombination. The *ppc* knock-out strains were selected by the LB agar plate containing 25 µg/mL kanamycin and verified by the PCR technique using the primers PPCa-U and KANk2- k2 (Table 2). The kanamycin-resistant gene in the chromosome was removed by introducing pCP20 [16] that allowed the expression of FLPase to catalyze the DNA recombination on the FRT site. More detail can be found in [8,9,15]. The construction of CA was achieved by deleting *pta* from the chromosome of CC using the procedure described above. Primers pta-HP1 and pta-HP2 were used to amplify FRT-*kan*^R^-FRT DNA fragment from pKD13 [16]. Primers acka-pta-U-new and KANk2- k2 were used to verify the in-frame insertion of FRT-*kan*^R^-FRT in CA.

### 2.2. Media and Cultivations

All *E. coli* strains used for fermentation studies were grown anaerobically in fresh 200 mL M9 mineral salts containing 20 g/L glucose and 2 g/L yeast extract. An anaerobic culture environment was achieved by using serum bottles sealed with butyl rubber stoppers and aluminum seals. The headspace of the sealed serum bottles was purged with filter-sterilized nitrogen for 10 min before the inoculation where the initial OD_600_ was adjusted to 0.05 [17]. Each bacterial culture was grown at 37 °C on a rotary incubator at 200 rpm. The pH of bacterial culture was adjusted to 8 at the fermentation times of 0, 8, and 24 h. The respective concentrations of streptomycin, chloramphenicol, and kanamycin used were 50, 34, and 50 μg/mL. Isopropyl-β-D-1-thiogalactopyranoside (IPTG) was added at 8 h to the final concentration of 0.02 mM when needed.

### 2.3. Analytical Methods

The cell density was measured at 600 nm using a UV-Vis spectrophotometer (GENESYS 10S, Thermo Scientific, Waltham, MA, USA). The measurement of gaseous CO_2_ concentration and the calculation of total CO_2_ concentration of a batch culture has been described in [7,8]. The characterization and quantification of glucose, formate, acetate, ethanol, succinate, pyruvate, and lactate were performed by using Thermo ScientificTM DionexTM Ulitmate 3000 LC Systems. The separation of the mixture was achieved with the Transgenomic ICSep ORH-801 column (300 mm × 6.5 mm, Transgenomic Inc., New Haven, CT, USA) where the measurement was done with a refractive index detector (for glucose and ethanol) and a UV-Vis detector (for the formate, acetate, succinate, pyruvate, and lactate). The mobile phase was 5 mM H_2_SO_4_. The column temperature was maintained at 45 °C while the flow rate was maintained at 0.6 mL/min. All samples were centrifuged for 5 min at 17,000× *g* to remove cell pellets and then supernatants were filtered by 0.2 μm PVDF filter before injection. The injection was done by an autosampler and the injection volume was 10 μL.

## 3. Results

### 3.1. Phenotypes of E. coli Strains CC and CA

**Figure 1:**
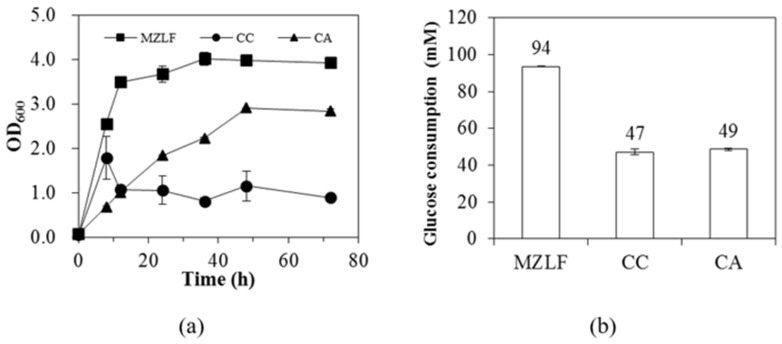
(**a**)The growth curve and (**b**) glucose consumption of *E. coli* strains MZLF, CC, and CA. M9 minimal salts with 111 mM glucose and 2 g/L yeast extract was used. The standard deviation was used for the error with n (biological replicates) ≥ 3. The genotypes of *E. coli* MZLF, CC, and CA are *E. coli* BL21(DE3) Δzwf, Δldh, Δfrd, MZLF (Δ*ppc*), and MZLF (Δ*ppc*, Δ*pta*). The genes *ppc* and *pta* encodes phosphoenolpyruvate carboxylase and phosphate acetyltransferase, respectively.

Phosphoenolpyruvate carboxylase (encoded by *ppc*) is an enzyme that converts phosphoenolpyruvate to oxaloacetate, which will be later used for the synthesis of the essential molecules for bacterial growth [18,19]. In this study, the deletion of *ppc* was lethal for the growth of strain CC in M9 minimal salts containing 111 mM glucose (data not shown), indicating that the *ppc* route is essential for the growth of *E. coli* CC when glucose is the sole carbon source. This is consistent with a previous study that *E. coli* CC (Δ*ppc*) cannot grow in a defined medium without the supplement of glutamate [13]. The supplement of yeast extract rescued the growth of strain CC and the OD_600_ of strain CC reached 1.8 ± 0.5 in 8 h and maintained around 1.0 throughout the whole cultivation (Figure 1a). The final glucose consumption of strain CC was 47 ± 2 mM, which was half of that of the parental strain MZLF (Figure 1b).

*E. coli* CA was constructed by deleting the *pta* gene (encodes phosphate acetyltransferase) from strain CC. Despite low growth in the first 12 h, OD_600_ of strain CA can reach 2.9 ± 0.0 in 48 h (Figure 1a), which was significantly higher than the parental strain CC. The glucose consumption of strain CA was 49 ± 1 mM at 36 h (Figure 1b), which was comparable to that of parental strain CC. The deletion of *pta* significantly decreased the acetate production (see results below), which consequently trimmed down the source of ATP production. The enhanced glucose consumption for strain CA can be attributed to the compensation of the ATP deficit and this enhancement in the carbon flux through the Embden–Meyerhof–Parnas (EMP) pathway may lead to the increase in the bacterial growth, see results below. Note that strain MZLF had a C-2/C-1 of 0.95 while strain CC had a C-2/C-1 of 0.94 -, where C-2 represents ethanol and acetate yields whereas C-1 represents formate and CO_2_ yields [9]. The insignificant difference of the C-2/C-1 between strains MZLF and CC indicates that the fixation of CO_2_ through the *ppc* route is negligible. The data further strengthen the validity of the index of C-2/C-1 to evaluate the efficiency of CO_2_ recycling by the Rubisco-based engineered pathway at the system level [9]. Table 3 listed glucose consumptions and fermentation product yields of bacterial strains tested in this study.

### 3.2. The Presence of PrkA Further Creates the Demand for ATP

**Figure 2:**
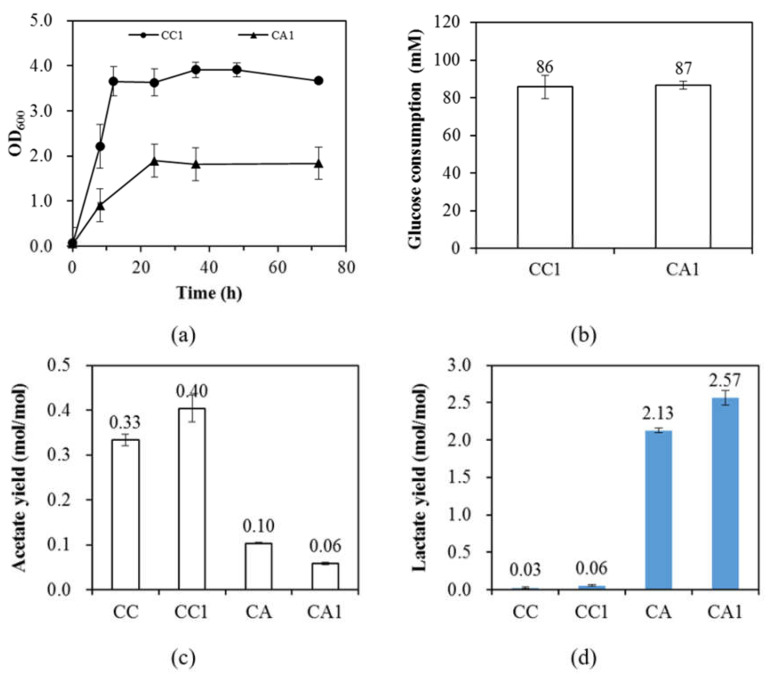
(**a**)The growth curve and (**b**) glucose consumption of *E. coli* strains CC1 and CA1. (**c**) The acetate and (**d**) lactate yields of *E. coli* strains CC, CC1, CA, and CA1. M9 minimal salts with 111 mM glucose and 2 g/L yeast extract was used. The standard deviation was used for the error with n (biological replicates) ≥ 3. The genotypes of CC and CA are MZLF (Δ*ppc*), and MZLF (Δ*ppc*, Δ*pta*), respectively. The nomenclature 1 of CC1 and CA1 represents strains CC and CA harboring P_BAD_-his6-*prkA*-pACYC184. The genes *ppc* and *pta* encodes phosphoenolpyruvate carboxylase and phosphate acetyltransferase, respectively.

In this study, it has been shown that PrkA can significantly enhance the growth of *E. coli* CC. It can be seen in Figure 2a that while strain CC reached OD_600_ of 0.82±0.08 at 36 h, PrkA further enhanced the growth of strain CC1 to OD_600_ of 3.91 ± 0.17 at 36 h. Meanwhile, the glucose consumption of strain CC1 was significantly increased from 47 ± 2 to 86 ± 6 mM compared to strain CC. (Figure 2b). Consistently, PrkA also enhanced the glucose consumption of strain CA1, where the glucose consumption of strain CA1 was 87 ± 2 mM (Figure 2b). Therefore, the increased glucose consumption is suggested to compensate for the need of ATP created by PrkA. In other words, PrkA enhanced the carbon flux through the EMP pathway in strain CC1 due to the demand for ATP. Note that the expression of PrkA is under control of P_BAD_ promoter which is known for its tight regulation. However, long lag phases were observed without the addition of *L*-arabinose in many circumstances as reviewed in the Introduction, indicating that *E. coli* is very sensitive to PrkA, even though the transcription of prkA has been well controlled by the tight P_BAD_ promoter.

The demand for ATP created by the expression of PrkA can also be evidenced by the change of acetate production. The acetate yield of strain CC1 was 0.40 ± 0.03 (*mol/mol*), which was 21% higher than that of strain CC (Figure 2c). The acetate productions of strains CA and CA1 were much lower than those of CC strains, indicating that *pta* is the major route for the acetate production. The decrease in the acetate productions indicates that the ATP supply in strains CA and CA1 cannot be obtained from the *pta* route. Meanwhile, it is suggested that the minor acetate production in strains CA and CA1 came from the pyruvate oxidation pathway (*poxB*) [20,21]. Since the acetate production derived from the *poxB* route provided no ATP production, the ATP-demanding PrkA did not stimulate acetate production in strain CA1, see Figure 2c. To compensate for the loss of ATP production due to the *pta* knockout, it is suggested that the EMP pathway becomes the major metabolic pathway for ATP production. Furthermore, the glucose consumption of CA1 was dramatically enhanced to 87 ± 2 mM compared to strain CA, see Figure 2b. It can be concluded that the deletion of *pta* can enhance the significance of the EMP pathway for ATP production and the enhancement can be greatly improved by the presence of PrkA. Note that intracellular ATP/ADP ratio can be correlated with the glycolytic flux where the glycolytic flux negatively responds to ATP/ADP ratio [22].

To track the overproduction of the reducing power due to the enhanced glycolysis through the EMP pathway, it was found that the lactate production was dramatically increased from 0.03 ± 0.01 to 2.13 ± 0.03 (*mol/mol*) when compared strains CA to CC (see Figure 2d). Interestingly, the presence of PrkA in strain CA1 further increased the lactate production from 2.13 ± 0.03 to 2.57 ± 0.10 (*mol/mol*). The results strongly support that the demand for ATP created by the *pta* deletion and the expression of PrkA enhanced glycolysis through the EMP pathway. The enhancement was accompanied by the overproduction of the reducing power and thus results in the activation of an auxiliary pathway that is responsible for the lactate accumulation. Note that the auxiliary pathway is suggested to be the *mgsA* (encodes methylglyoxal synthase) route (the methylglyoxal pathway) [23,24,25,26,27]. The high lactate production of strain CA1 with a yield of 2.57 ± 0.10 mol/mol-glucose suggests that the carbon source was derived from both supplemented glucose and yeast extract. The lactate production was primarily derived from glucose consumption since the glucose consumption of strain CA1 was 87 ± 2 mM, which was far larger than the yeast extract concentration (2 g/L) that was supplemented. It also suggests that the internal reducing power was highly overproduced and may adversely affect the growth of strain CA1 compared to strain CA. Note that no significant long lag phase was observed for strains CC1 and CA1 (Figure 2a), which is different from previous studies as discussed above. This suggests that the implementation of physiological stresses should reveal the hidden impact of heterologous expression of PrkA on microbial physiology. In this study, PrkA is found to enhance glucose consumption. This is consistent with previous literature that *E. coli* can find a way to detoxify the adverse effect caused by PrkA [7].

### 3.3. The Overexpression of Rubisco Strongly Directs Carbon into Biomass and Ethanol Production

**Figure 3:**
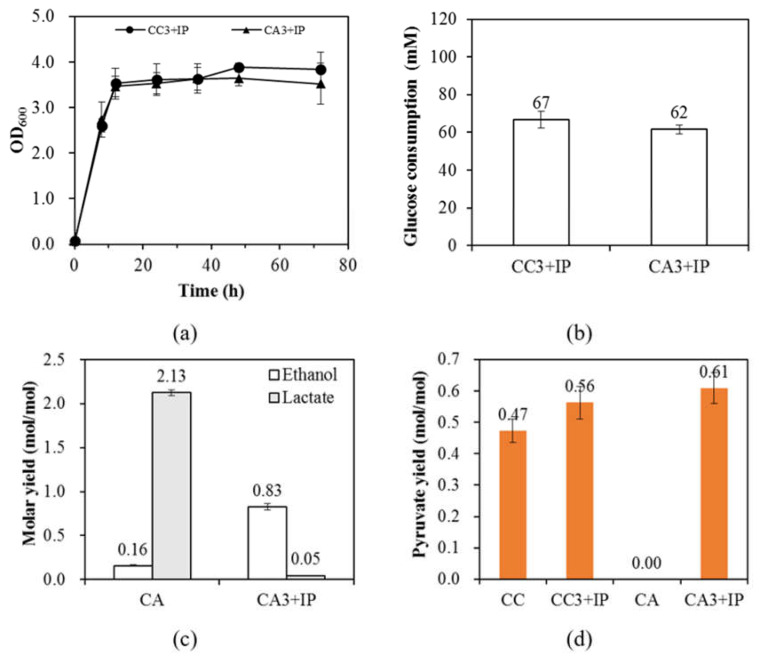
(**a**)The growth curve and (**b**) glucose consumption of *E. coli* strains CC3 + IP and CA3 + IP. (**c**) The ethanol and lactate yields of *E. coli* strains CA and CA3 + IP and (**d**) the pyruvate yield of CC, CC3 + IP, CA, and CA3 + IP. M9 minimal salts with 111 mM glucose and 2 g/L yeast extract was used. CC3 + IP and CA3 + IP represent the addition of 0.02 mM IPTG to induce the expression of Rubisco in CC3 and CA3, respectively. The standard deviation was used for the error with *n* (biological replicates) ≥ 3. The genotypes of CC and CA are MZLF (Δ*ppc*), and MZLF (Δ*ppc*, Δ*pta*), respectively. The nomenclature 3 of CC3 and CA3 represents strains CC and CA harboring *rbcLS*-pET30a + (M259T). IP represents the addition of 0.02 mM IPTG to induce the expression of Rubisco in CC3 and CA3. The genes *ppc* and *pta* encodes phosphoenolpyruvate carboxylase and phosphate acetyltransferase, respectively.

Figure 3a showed that the overexpression of Rubisco greatly enhanced the growth of strains CC3 + IP and CA3 + IP and both strains exhibited faster growth rates and higher final OD_600_. The better growths of strains CC3 + IP and CA3 + IP were accompanied by enhanced glucose consumption (Figure 3b). Strains CC and CA had a glucose consumption of 47 ± 2 and 49 ± 1 mM whereas strains CC3 + IP and CA3 + IP had 67 ± 5 and 62 ± 2 mM, respectively. While the enhanced bacterial growth due to the overexpression of Rubisco is consistent with previous literature [9], the enhanced glucose consumption shown in Figure 3b proves the assumption made in [9] that the overexpression of Rubisco also enhances the glycolysis through the EMP pathway. This enhancement can be fully revealed when the intracellular energy balance is further disrupted by deleting *pta*. Note that strains CC3 + IP and CA3 + IP represent the addition of 0.02 mM IPTG to induce the expression of Rubisco in strains CC3 and CA3, respectively.

Another result about Rubisco is that it changed the way to balance the reducing power in *E. coli* where strain CA3 + IP favored the production of ethanol rather than lactate. This can be seen in Figure 3c that the production of ethanol in strain CA3 + IP significantly increased from 0.16 ± 0.01 to 0.83 ± 0.04 (*mol/mol*) while the lactate yield decreased from 2.13 ± 0.03 to 0.05 ± 0.00 (*mol/mol*). The transition from lactate production to ethanol production suggests that Rubisco strongly favors the production of acetyl-CoA for the need of the glyoxylate shunt where the regeneration of NAD^+^ is achieved by ethanol production. The high activity of ethanol production was accompanied by the accumulation of pyruvate. It can be seen in Figure 3d that pyruvate production was increased from nothing to 0.61 ± 0.05 mol/mol when Rubisco was overexpressed in strain CA3 + IP, which was positively correlated with the ethanol production. Since Rubisco alone is sufficient to induce the results as shown in Figure 3, it is believed that it is the expression of Rubisco to induce the physiological responses rather than the function of Rubisco (since there was no substrate for Rubisco). This is consistent with our study where the Rubisco mutant can also induce the accumulation of pyruvate in *E. coli* MZLF [9]. Note that Figure 3d showed that Rubisco enhanced pyruvate production in both strains CC3 + IP and CA3 + IP.

## 4. Discussion

**Figure 4:**
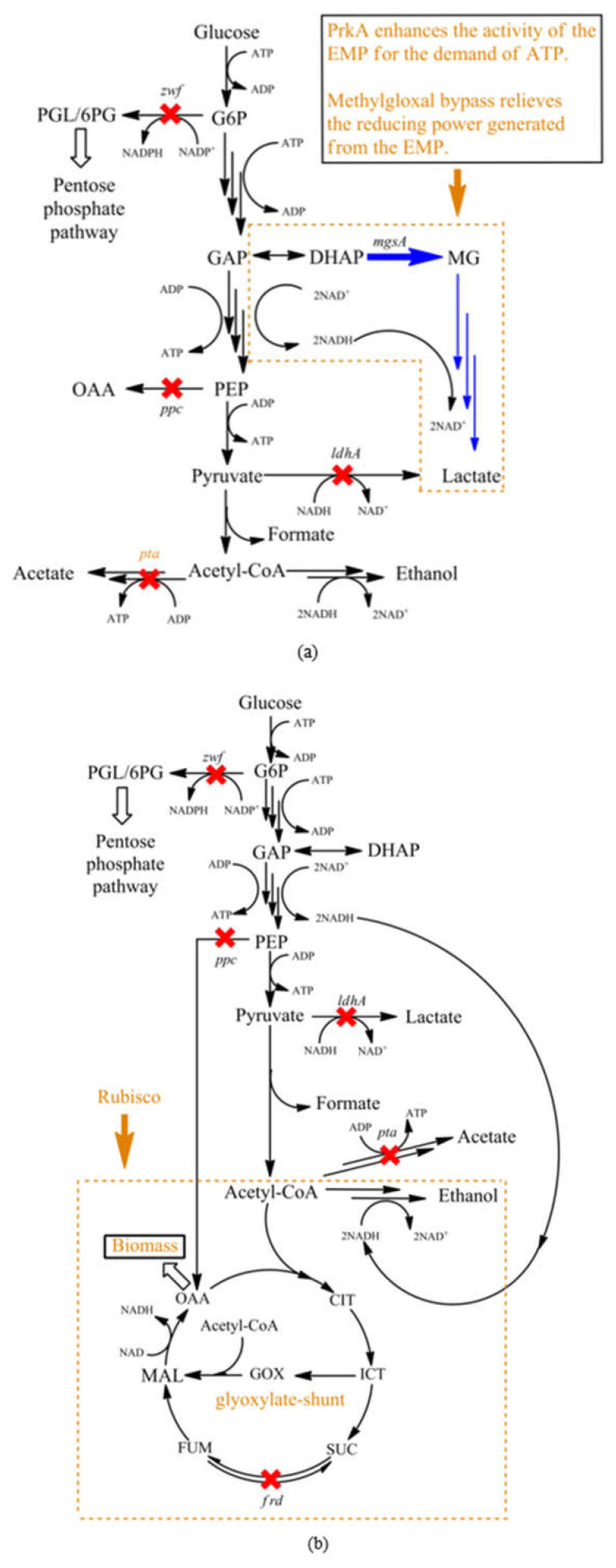
Proposed models for (**a**) PrkA-induced metabolic shift and (**b**) Rubisco-induced glyoxylate shunt. Abbreviations: [CIT], citrate; [DHAP], dihydroxyacetone phosphate; [FUM], fumarate; [GAP], glyceraldehyde 3-phosphate; [GOX], glyoxylate; [G6P], glucose 6-phosphate; [ICT], isocitrate; [MAL], malate; [MG], methylglyoxal; [OAA], oxaloacetate; [PEP], phosphoenolpyruvate; [6PG], 6-phosphogluconate; [PGL], 6-phosphoglucono-lactone; [SUC], succinate.

*E. coli* strains CC and CA were constructed to fully reveal Rubisco- or PrkA-induced physiological impacts. The stress for the cell growth created by *ppc* deletion can be demonstrated by the non-growth of CC in M9 minimal salts containing 111 mM glucose (data not shown). Furthermore, the glucose consumption of CC3 + IP (2YE) was only half of MZLF3 + IP (0YE) demonstrated that *ppc* knockout effectively weakened the activity of the EMP pathway. On the other hand, we have shown that both PrkA and Rubisco enhances glycolysis through the EMP pathway. Unlike the restricted growth of strain CA1 (Figure 3c), the enhanced bacterial growths of strains CC3 + IP and CA3 + IP were accompanied along with the enhanced EMP activity (Figure 3a and b). It is believed that the enhanced EMP activity is to justify the ATP demand created by PrkA, Rubisco, and the *pta* knockout. Therefore, the enhanced EMP activity led to the overproduction of the reducing power. Interestingly, PrkA and Rubisco induced different metabolic pathways to relieve it. PrkA may induce the *mgsA* route so that lactate was the major product (Figure 2d). On the other hand, Rubisco adopted the traditional ethanol fermentation route to reach the redox balance in strain CA, even though *pta* was disrupted (Figure 3c). This indicates that Rubisco may induce a high demand for acetyl-CoA which is subsequently used by the glyoxylate shunt. This hypothesis is consistent with not only the enhanced growth of strain CA3 (Figure 3a) but also previous studies that Rubisco can enhance the bacterial growth in different *E. coli* strains and cultivation conditions [7,9]. The importance and competitiveness of the glyoxylate shunt have been previously proposed. The glyoxylate shunt has been computationally shown as a competent route to respond to the stress caused by the *ppc* knockout in *E. coli* MG1655 [12]. The glyoxylate shunt also computationally shown its competitive role in cyanobacteria to reach a stable metabolic network, even its existence in cyanobacteria has been fully proved [28,29]. In this study, the glyoxylate shunt has been proposed to respond to the metabolic perturbation caused by the overexpression of Rubisco. It is well known that the metabolic network will thermodynamically evolve itself to genetic stability to respond to sorts of perturbations, where the evolution may take hundreds of generations [12]. Meanwhile, each component of a metabolic network also has different kinetics to respond to perturbations [30]. We further interrogate a possibility that there is a hierarchy in *E. coli* to handle the redox balance where the *mgsA* route may only be adopted for the severe redox imbalance. At least the *mgsA* route showed no kinetics competitiveness when redox imbalance is relatively not severe due to the mild glucose consumption. Figure 4a and b show the proposed models for PrkA- and Rubisco-induced metabolic shifts, respectively.

## 5. Conclusions

The enhanced glycolysis through the EMP pathway as well as enhanced auxiliary pathways become a competition for the Rubisco engineered pathway to in situ recycle CO_2_. This study showed that heterologous expression of PrkA or Rubisco perturbed the physiology of *E. coli* by changing its internal energy balance and carbon flow, which were not favored for in situ CO_2_ recycling by the Rubisco-based engineered pathway. Both PrkA and Rubisco enhances the glycolysis through the EMP pathway. Together with the enhanced biomass, Rubisco is suggested to induce a high demand for acetyl-CoA which is subsequently used by the glyoxylate shunt.

## Figures and Tables

**Table 1:** The list of bacterial strains and plasmids.

Name	Descriptions	Reference
**Bacterial strains**		
*E. coli* BL21 (DE3)	F-, *dcm*, *ompT*, *gal*, *lon*, *hsd*S_B_(rB^-^, mB^-^), λ(DE3[*lac*I, *lac*UV5-T7 gene 1, *ind*1, *sam*7,*nin*5])	A gift from Prof. Huang, Chieh-Chen at NCHU, Taiwan
MZLF	*E. coli* BL21(DE3) Δ*zwf*, Δ*ldh*, Δ*frd*	[9]
CC	*E. coli* BL21(DE3) Δ*zwf*, Δ*ldh*, Δ*frd*, Δ*ppc*	This study
CC1	*E. coli* BL21(DE3) Δ*zwf*, Δ*ldh*, Δ*frd*, Δ*ppc* harboring P_BAD_-his6-*prkA*-pACYC184	This study
CC3	*E. coli* BL21(DE3) Δ*zwf*, Δ*ldh*, Δ*frd*, Δ*ppc* harboring *rbcLS*-pET30a + (M259T)	This study
CA	*E. coli* BL21(DE3) Δ*zwf*, Δ*ldh*, Δ*frd*, Δ*ppc*, Δ*pta*	This study
CA1	*E. coli* BL21(DE3) Δ*zwf*, Δ*ldh*, Δ*frd*, Δ*ppc*, Δ*pta* harboring P_BAD_-his6-*prkA*-pACYC184	This study
CA3	*E. coli* BL21(DE3) Δ*zwf*, Δ*ldh*, Δ*frd*, Δ*ppc*, Δ*pta* harboring *rbcLS*-pET30a + (M259T)	This study
**Plasmids**		
pKD46	*araC*, *bla*, *oriR101*, *repA*101(Ts), *araC-P_araB_*-*γ*-*β-exo* (encode λ Red recombinases), temperature-conditional replicon	[15]
pkD13	*bla*, FRT-*kan*-FRT	[15]
pCP20	*FLP*^+^, λ *c*I857 ^+^,λ P_R_ Pep^ts^, *bla*, *catF*	[16]
P_BAD_-his6-*prkA*-pACYC184	Recombinant plasmid carries *prkA* gene (derived from *Synechococcus* PCC7492) for the overexpresion of phosphoribulokinase (PrkA) under the control of P_BAD_	[6]
*rbcLS*-pET30a + (M259T)	Recombinant plasmid carries engineered *rbcLS* gene (originated from *Synechococcus* PCC6301) for the overexpresion of engineered Rubisco (M259T) under the control of P_T7_	[6]

**Table 2:** Primers used in this study.

Name	Sequence
PPChf-HP1	CGTGAAGGATACAGGGCTATCAAACGATAAGATGGGGTGTCTGGGGTAAT GTGTAGGCTGGAGCTGCTTC
PPChr-HP2	ATTTCAGAAAACCCTCGCGCAAAAGCACGAGGGTTTGCAGAAGAGGAAGAA TTCCGGGGATCCGTCGACC
PPCa-U	CGTGAAGGAT ACAGGGCTATC
KANk2- k2	CGGTGCCCTGAATGAACTGC
pta-HP1	ACACCGCCAGCTCAGCTGGCGGTGCTGTTTTGTAACCCGCCAAATCGGCGGTAACGAAAGAGGATAAACC GTGTAGGCTGGAGCTGCTTC
pta-HP2	TAAAAAACCGGAAATAGTGATTATTTCCGGTTCAGATATCCGCAGCGCAAAGCTGCGGATGATGACGAGAATTCCGGGGATCCGTCGACC
acka-pta-U-new	GTGTCATCATGCGCTACGCT

**Table 3:** Glucose consumptions and fermentation product yields of bacterial strains tested in this study. The standard deviation was used for the error with *n* (biological replicates) ≥ 3.

Strain ^1^		Fermentation Product Yield (*mol/mol*) ^2^							
	Glucose Consumption (mM)	Formate	Acetate	Ethanol	Succinate	Pyruvate	Lactate	CO_2_	Biomass
MZLF	94 ± 0	1.15 ± 0.00	0.37 ± 0.00	0.84 ± 0.00	0.009 ± 0.000	0.41 ± 0.00	0.13 ± 0.01	0.13 ± 0.02	0.62 ± 0.02
CC	47 ± 2	1.13 ± 0.06	0.33 ± 0.01	0.82 ± 0.06	0.006 ± 0.002	0.47 ± 0.04	0.03 ± 0.01	0.09 ± 0.05	0.25 ± 0.02
CC1	86 ± 6	1.20 ± 0.12	0.40 ± 0.03	0.95 ± 0.18	0.006 ± 0.001	0.46 ± 0.10	0.06 ± 0.01	0.10 ±0.01	0.66 ± 0.06
CC3 + IP	67 ± 5	1.06 ± 0.09	0.29 ± 0.03	0.76 ± 0.10	0.008 ± 0.002	0.56 ± 0.05	0.05 ± 0.01	0.04 ± 0.01	0.79 ± 0.09
CA	49 ± 1	0.27 ± 0.01	0.10 ± 0.00	0.16 ± 0.01	0.017 ± 0.000	0.00 ± 0.00	2.13 ± 0.03	0.09 ± 0.00	0.66 ± 0.01
CA1	87 ± 2	0.16 ± 0.00	0.06 ± 0.00	0.04 ± 0.00	0.003 ± 0.000	0.02 ± 0.00	2.57 ± 0.10	0.04 ± 0.00	0.30 ± 0.01
CA3 + IP	62 ± 2	1.16 ± 0.05	0.33 ± 0.02	0.83 ± 0.04	0.010 ± 0.002	0.61 ± 0.05	0.05 ± 0.00	0.04 ± 0.01	0.85 ± 0.07

^1^: CC3 + IP and CA3 + IP represent the addition of 0.02 mM IPTG to induce the expression of Rubisco in CC3 and CA3, respectively,^2^: The product yield is calculated based on the glucose consumption where the contribution of yeast extract is not taken into consideration.
